# Clofazimine-induced skin pigmentation

**DOI:** 10.11604/pamj.2024.47.86.42513

**Published:** 2024-02-26

**Authors:** Ashwin Karnan, Anjana Ledwani

**Affiliations:** 1 Department of Respiratory Medicine, Jawaharlal Nehru Medical College, Datta Meghe Institute of Higher Education and Research, Sawangi (Meghe), Wardha, Maharashtra, India

**Keywords:** Clofazimine, cough, tuberculosis, hyperpigmentation

## Image in medicine

A 54-year-old male presented to the outpatient department with complaints of black discoloration of skin and nails for the past 1 month. The patient gave a history of multi-drug resistant sputum positive tuberculosis for which he is on antitubercular drugs (Isoniazid, Ethambutol, Pyrazinamide, Clofazimine, Bedaquiline, Levofloxacin, and Ethionamide) for the past 9 months. A dermoscopy was done which showed yellow globules in a honeycombing pattern and hyperpigmentation with sparing of the sweat glands. A diagnosis of Clofazimine-induced hyperpigmentation was made. As a physician, with tuberculosis nearing elimination, monitoring and managing the adverse effects of drugs is important. Clofazimine is a riminophenazine dye that has both anti-microbial and anti-inflammatory action. It is absorbed orally and is known to accumulate in tissues. It has a very long half-life of about 70 days and is excreted in small amounts in bile, urine, and sweat. It is used in leprosy, multidrug-resistant tuberculosis, lepra reaction, and in other mycobacterial infections. Clofazimine-induced pigmentation is reversible but takes months to years to clear after stopping the drug.

**Figure 1:**
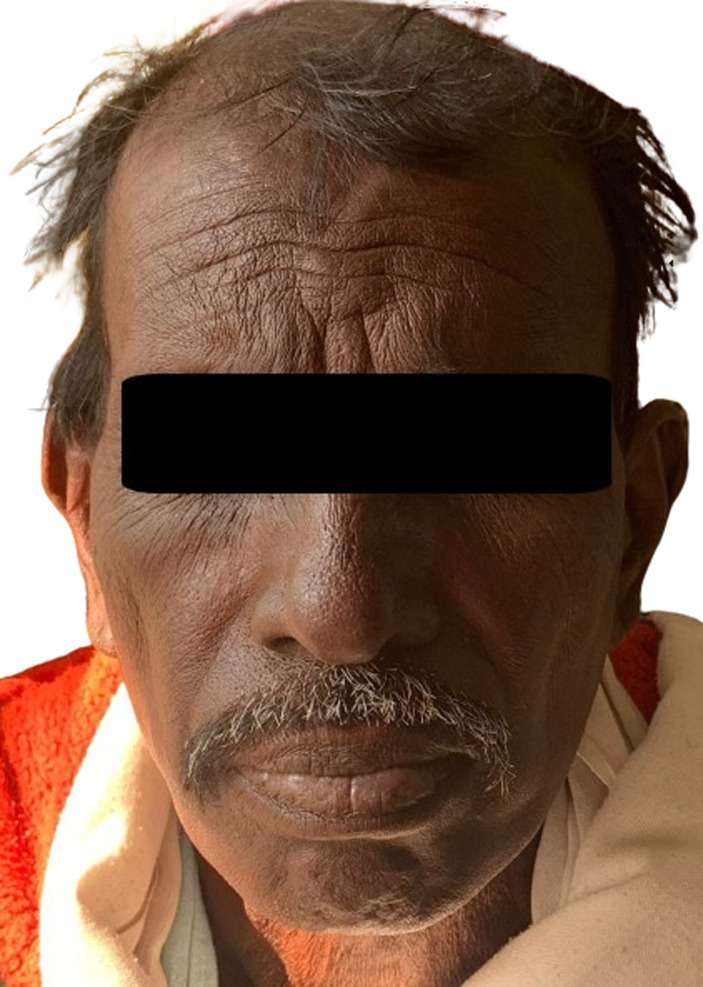
generalized hyperpigmentation over the face

